# T-Cell Epitopes Shared Between Immunizing HLA and Donor HLA Associate With Graft Failure After Kidney Transplantation

**DOI:** 10.3389/fimmu.2021.784040

**Published:** 2021-11-18

**Authors:** Emma T. M. Peereboom, Benedict M. Matern, Toshihide Tomosugi, Matthias Niemann, Julia Drylewicz, Irma Joosten, Wil A. Allebes, Arnold van der Meer, Luuk B. Hilbrands, Marije C. Baas, Franka E. van Reekum, Marianne C. Verhaar, Elena G. Kamburova, Marc A. J. Seelen, Jan Stephan Sanders, Bouke G. Hepkema, Annechien J. Lambeck, Laura B. Bungener, Caroline Roozendaal, Marcel G. J. Tilanus, Christien E. Voorter, Lotte Wieten, Elly M. van Duijnhoven, Mariëlle A. C. J. Gelens, Maarten H. L. Christiaans, Frans J. van Ittersum, Azam Nurmohamed, Neubury M. Lardy, Wendy Swelsen, Karlijn A. van der Pant, Neelke C. van der Weerd, Ineke J. M. ten Berge, Fréderike J. Bemelman, Aiko P. J. de Vries, Johan W. de Fijter, Michiel G. H. Betjes, Dave L. Roelen, Frans H. Claas, Henny G. Otten, Sebastiaan Heidt, Arjan D. van Zuilen, Takaaki Kobayashi, Kirsten Geneugelijk, Eric Spierings

**Affiliations:** ^1^ Center for Translational Immunology, University Medical Center Utrecht, Utrecht University, Utrecht, Netherlands; ^2^ Department of Transplant Surgery, Japanese Red Cross Aichi Medical Center Nagoya Daini Hospital, Nagoya, Japan; ^3^ Department of Kidney Diseases and Transplant Immunology, Aichi Medical University School of Medicine, Nagakute, Japan; ^4^ PIRCHE AG, Berlin, Germany; ^5^ Laboratory Medicine, Laboratory Medical Immunology, Radboud University Medical Center, Nijmegen, Netherlands; ^6^ Department of Nephrology, Radboud University Medical Center, Nijmegen, Netherlands; ^7^ Department of Nephrology, University Medical Center Utrecht, Utrecht University, Utrecht, Netherlands; ^8^ Division of Nephrology, Department of Internal Medicine, University Medical Center Groningen, University of Groningen, Groningen, Netherlands; ^9^ Department of Laboratory Medicine, University Medical Center Groningen, University of Groningen, Groningen, Netherlands; ^10^ Department of Transplantation Immunology, Tissue Typing Laboratory, Maastricht University Medical Center, Maastricht, Netherlands; ^11^ Department of Internal Medicine, Division of Nephrology, Maastricht University Medical Center, Maastricht, Netherlands; ^12^ Department of Nephrology, Amsterdam University Medical Center, Amsterdam, Netherlands; ^13^ Department of Immunogenetics/HLA Diagnostic, Sanquin Diagnostic Services, Amsterdam, Netherlands; ^14^ Renal Transplant Unit, Department of Internal Medicine, Amsterdam University Medical Center, Amsterdam, Netherlands; ^15^ Department of Nephrology, Leiden University Medical Center, Leiden, Netherlands; ^16^ Department of Internal Medicine, Erasmus MC, Rotterdam, Netherlands; ^17^ Department of Nephrology, Erasmus MC, Rotterdam, Netherlands; ^18^ Department of Immunology, Leiden University Medical Center, Leiden, Netherlands; ^19^ Department of Renal Transplant Surgery, Aichi Medical University School of Medicine, Nagakute, Japan

**Keywords:** HLA antigens, PIRCHE-II, graft failure, kidney transplantation, shared T-cell epitopes, T-cell epitope, T-cell memory

## Abstract

CD4^+^ T-helper cells play an important role in alloimmune reactions following transplantation by stimulating humoral as well as cellular responses, which might lead to failure of the allograft. CD4^+^ memory T-helper cells from a previous immunizing event can potentially be reactivated by exposure to HLA mismatches that share T-cell epitopes with the initial immunizing HLA. Consequently, reactivity of CD4^+^ memory T-helper cells toward T-cell epitopes that are shared between immunizing HLA and donor HLA could increase the risk of alloimmunity following transplantation, thus affecting transplant outcome. In this study, the amount of T-cell epitopes shared between immunizing and donor HLA was used as a surrogate marker to evaluate the effect of donor-reactive CD4^+^ memory T-helper cells on the 10-year risk of death-censored kidney graft failure in 190 donor/recipient combinations using the PIRCHE-II algorithm. The T-cell epitopes of the initial theoretical immunizing HLA and the donor HLA were estimated and the number of shared PIRCHE-II epitopes was calculated. We show that the natural logarithm-transformed PIRCHE-II overlap score, or Shared T-cell EPitopes (STEP) score, significantly associates with the 10-year risk of death-censored kidney graft failure, suggesting that the presence of pre-transplant donor-reactive CD4^+^ memory T-helper cells might be a strong indicator for the risk of graft failure following kidney transplantation.

## Introduction

Kidney transplantation is the preferred treatment option for many patients with end-stage kidney disease. The outcome of such a treatment is most optimal when recipient and donor are human leukocyte antigen (HLA) matched (1, 2). Through the activation of alloimmune T and B cells, HLA mismatches may lead to T-cell-mediated rejection, or, via the development of donor-specific HLA antibodies (DSA), to antibody-mediated rejection (3). While treated T-cell-mediated rejection is not associated with reduced graft survival (4, 5), antibody-mediated rejection still often leads to graft failure and is currently even the leading cause of late kidney graft failure (4, 6–8).

The degree of HLA matching at the antigen level has been observed to be associated with graft survival (9). However, several studies have shown that epitope-based HLA matching could be more effective than antigen-based HLA matching (10–14), as antigen-based HLA matching has some limitations. For example, a mismatched HLA allele may lack immunogenic epitopes capable of inducing an alloimmune response. Only epitopes that are specific for the mismatched HLA of the donor, and absent in HLA of the recipient, can potentially elicit an alloimmune response (15). Consequently, various algorithms have been developed for prediction of HLA matching based on B-cell epitopes, such as HLA‐EMMA and HLAMatchmaker (16, 17).

CD4^+^ T-helper cells are of significant importance during allograft rejection. During T-cell-mediated rejection, these cells are likely able to stimulate intermediary antigen-presenting cells, which can consequently prime CD8^+^ cytotoxic T-cells (18, 19). In the formation of HLA antibodies, CD4^+^ T-helper cells can stimulate B-cell proliferation and differentiation. More specifically, CD4^+^ T-helper cells may recognize epitopes derived from mismatched HLA via the indirect pathway of allorecognition (20, 21). In this process of indirect allorecognition, mismatched HLA molecules are internalized and processed into peptide fragments by antigen-presenting cells such as B cells. These HLA-derived peptides are subsequently presented on the cell surface of these antigen-presenting cells by HLA class II molecules. The CD4^+^ T-helper cells, in turn, recognize these allopeptide-HLA class II complexes and provide help to naive B cells by stimulating B-cell proliferation, differentiation of naive B cells into antibody-producing plasma cells and memory B cells, and immunoglobulin class switching (22–24). Thus, these CD4^+^ T-helper cells may contribute to the formation of *de novo* DSA (25–27).

According to the immunological concept of linked recognition, CD4^+^ T-helper cells and B cells should recognize individual epitopes that are derived from the same mismatched HLA molecule (28, 29). Following this concept, the PIRCHE-II (Predicted Indirectly ReCognizable HLA Epitopes presented by recipient HLA class II) algorithm has recently been developed as a T-cell epitope-based risk stratification in transplantation (15). The PIRCHE-II algorithm estimates the number of mismatched HLA-derived peptides that are absent in the recipient and that can be presented by the HLA class II molecules of the recipient. The number of PIRCHE-II for each donor-recipient combination reflects the theoretical potential level of CD4^+^ T-cell alloreactivity (15). Studies have shown that increasing PIRCHE-II numbers associate with *de novo* development of DSA (30–33) and reduced kidney graft survival (31, 32).

The presence of donor-specific CD4^+^ memory T cells at the time of transplant may compromise the transplant outcome, since these cells can lead to a rapid production of antibodies as compared to naive alloimmune T-helper cells (34). Although the frequency of IFNγ-producing donor-reactive memory T cells in patients can be quantified using enzyme-linked ImmunoSpot (ELISPOT) assays (35–38), the detection of pre-transplant donor-reactive CD4^+^ memory T cells remains challenging. As an alternative, the presence of shared T-cell epitopes could be assessed. Following the primary T-cell immune response, memory T cells can be reactivated by rechallenging them with their T-cell epitope. This T-cell epitope may be derived from the initial immunizing HLA antigen, or alternatively, this epitope could be shared between the initial HLA mismatch (designated as immunizing HLA) and the mismatched HLA molecules present in the new transplant (designated as donor HLA). Consequently, the presence of T-cell epitopes that are shared between immunizing and donor HLA could potentially increase the risk of alloimmune reactions following transplantation. These shared T-cell epitopes may explain why recipients with pre-transplant non-donor-specific HLA antibodies experience have a decreased graft survival (39) and diminished graft function (40) as compared to patients without these HLA antibodies. A recent study investigated the effect of shared T-cell epitopes on the development of *de novo* DSA using the PIRCHE-II algorithm (41). Patients theoretically able to recognize shared T-cell epitopes had a higher incidence of early *de novo* DSA formation compared to patients without shared T-cell epitopes, suggesting that T-cell epitopes shared between immunizing and donor HLA indicate a significant risk of early *de novo* DSA development after transplantation (41).

Although the effect of shared T-cell epitopes on *de novo* DSA development has been studied (41), the effect of shared T-cell epitopes on graft failure remains uninvestigated. In the current study, we therefore evaluated the role of these shared T-cell epitopes in the context of kidney graft failure. To do so, the potential T-cell epitopes originating from the theoretical immunizing HLA and donor HLA were determined using the PIRCHE-II algorithm. Subsequently, the number of T-cell epitopes shared between immunizing and donor HLA was calculated as a surrogate marker to estimate the potential of CD4^+^ T-helper cell recall responses. Here, we hypothesize that recipients with a higher number of overlapping PIRCHE-II peptides between immunizing and donor HLA have an increased risk of developing graft failure as compared to recipients with a lower number of overlapping PIRCHE-II peptides.

## Materials and Methods

### Study Population

**Figure 1:**
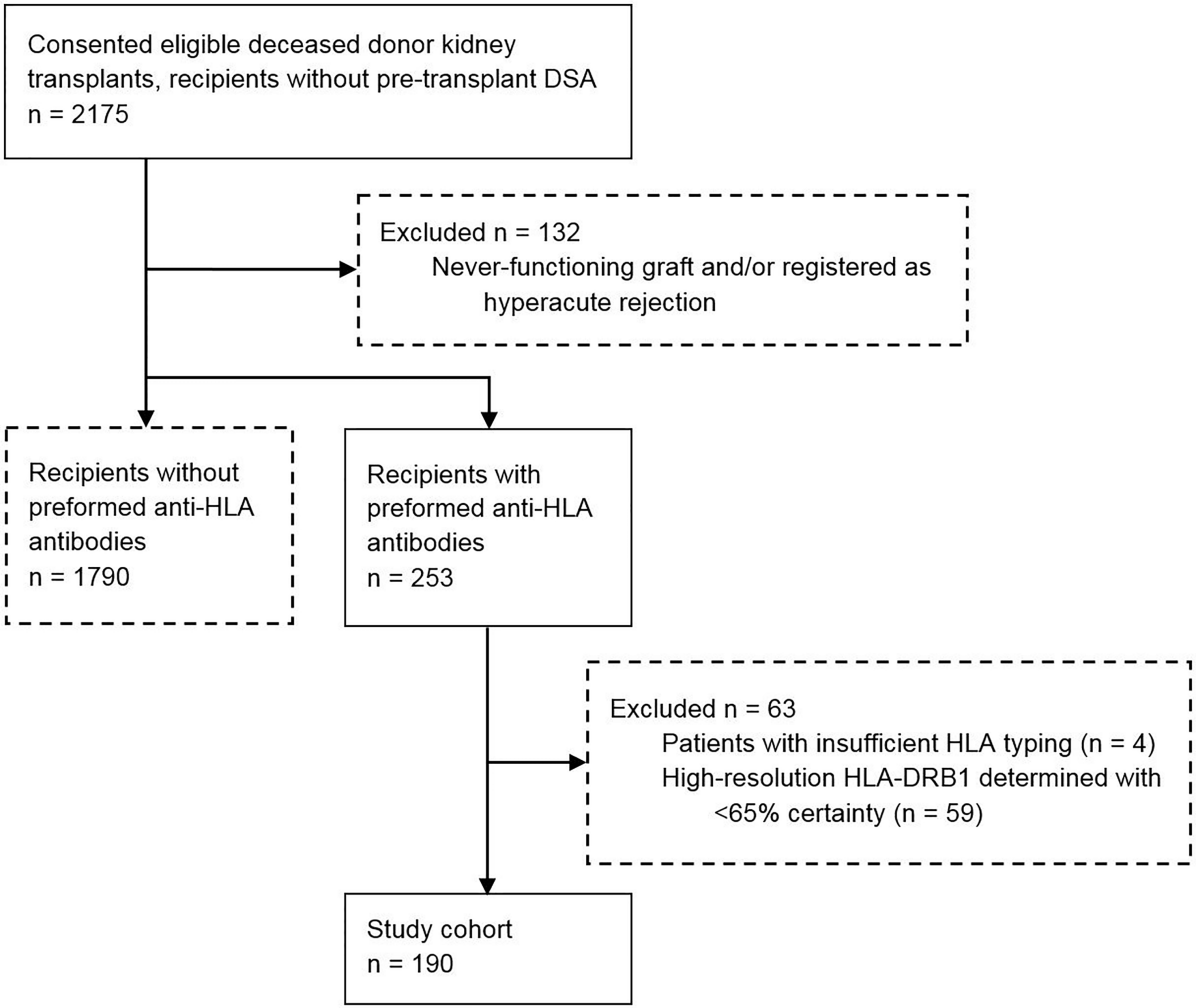
Inclusion and exclusion criteria. Boxes with dotted lines represent excluded recipients.

In the current retrospective multicenter study, we evaluated 2175 kidney transplant recipients from the PROCARE cohort, a cohort which included all deceased donor kidney transplantations performed in the seven transplantation centers in the Netherlands between 1995 and 2005 (42). All transplants were performed after a negative T-cell complement dependent cytotoxicity (CDC) crossmatch. Pre-transplant sera were drawn from all recipients and retrospectively assessed for the presence of HLA antibodies as previously described (43). This study included only recipients who did not have pre-transplant DSA. All in- and exclusion criteria have been listed in **Figure 1**. In total, 190 transplant recipients with pre-transplant non-DSA HLA antibodies were included in this study. HLA typing was available for all included kidney transplant donors and recipients at serological level for a minimum of HLA-A, -B, and -DR. HLA-C and -DQ typing of donors and recipients were included in the dataset when available. Clinical data including data regarding graft failure, death with a functioning graft, and follow-up time were obtained from the Dutch Organ Transplant Registry. Detailed methods on data collection of the PROCARE cohort have been described previously (43). Patients were followed up at 3 months, 12 months, and yearly thereafter for at least 10 years. Graft failure was defined as loss of kidney function when the patient returned to dialysis or received a repeat transplant (43). The etiology of graft failures was not documented. Informed consent was obtained from all participants. The study was approved by the ethics committee for biobanks at the University Medical Center Utrecht (TCBio; reference number: 13-633).

### Identification of Immunizing HLA

For each recipient with HLA antibodies but no pre-transplant DSA, the theoretical immunizing HLA allele was identified based upon the documented response using a Luminex Single Antigen beads (LSA) assay (Immucor, Stamford, CT) according to the manufacturer’s instructions (44). Raw mean fluorescence intensity (MFI) values were corrected by dividing the MFI value of each bead by the MFI of the lowest ranked antigen (LRA) per recipient per locus (HLA-A, -B, -C, -DR1/3/4/5, -DQA1/DQB1, and -DPA1/DPB1) (MFI/LRA MFI) (45).

### Identification of PIRCHE-II Peptides

The PIRCHE-II peptides originating from the initial theoretical immunizing HLA alleles (‘immunizers’) and the PIRCHE-II peptides originating from the donor’s HLA alleles serving as potential target for the recipient (‘recall epitopes’) were identified using the PIRCHE-II algorithm version 3.3.30 (PIRCHE AG, Berlin, Germany, available *via* www.pirche.com). HLA-A, -B, -C, -DRB1, -DRB3/4/5, -DQA1/DQB1, and -DPA1/DP1 were taken into consideration as presented loci, and HLA-DRB1 was considered as presenting locus. As only HLA typing data at serological level were available in our cohort, the PIRCHE-II peptides and their weights were calculated based on serological typing data as described previously (46). In short, the most likely high-resolution HLA genotypes for each serological HLA typing were identified using HLA haplotype frequency tables from the National Marrow Donor Program of 2007 (47). For each high-resolution HLA genotype, the PIRCHE-II peptides were calculated and weighted against the normalized frequency of the high-resolution HLA haplotypes in the general population. Four patients were excluded from further analyses due to insufficient HLA typing that did not allow extrapolation to high-resolution HLA genotypes (**Figure 1**).

Since serological typing of the presenting locus HLA-DRB1 might hamper the identification of overlapping T-cell epitopes, the serological HLA-DR typing of the recipients was extrapolated to high resolution using the PIRCHE-II algorithm as described previously (46). In short, using the serological HLA typing, all possible high-resolution HLA-DRB1 alleles within the same recipient with their weights - representing the likelihood of a specific high-resolution typing – were calculated. When the high-resolution HLA-DRB1 allele with the highest calculated weight had a weight of at least 0.65 (with a maximum of 1), the serological HLA-DR was replaced by this high-resolution HLA-DRB1. When a recipient did not have a high-resolution HLA-DRB1 allele with a weight of at least 0.65, the recipient was excluded from further analyses, as the selection of an incorrect high-resolution HLA-DRB1 would affect the PIRCHE-II calculations of both the immunizing and donor HLA. An example of the extrapolation from serological HLA-DR to high-resolution HLA-DRB1 typing is provided in **Supplementary Table 1**. In total, from the 253 patients with HLA antibodies, 59 recipients were excluded from further analyses (**Figure 1**). The ExtrapolateDRB1 script used to identify the high-resolution HLA-DRB1 for each recipient is available on https://github.com/EPeereboom/PIRCHE-II-Overlap/blob/main/ExtrapolateDRB1.py.

The PIRCHE-II peptides involved in the generation of the HLA antibodies were calculated as described previously (33), using the HLA typing of the recipient and the LSA-identified immunizing HLA allele as input for the PIRCHE-II algorithm. To identify these immunizing HLA alleles, only the positive beads from the pre-transplant LSA were used. To this end, four approaches were evaluated to select the LSA-positive alleles: (A) Inclusion of the beads with an MFI/LRA MFI ratio above a threshold of 5; (B) Inclusion of the bead(s) with the highest positive MFI for each recipient, following the approach of Tomosugi et al. (41); (C) Inclusion of the beads with an MFI/LRA MFI ratio in the upper 5% range of MFI/LRA MFI ratios for each recipient using the formula cutoffRatio = minRatio + (maxRatio – minRatio)*(1 – 0.05); (D) Inclusion of the beads with an MFI/LRA MFI ratio in the upper 1% range of MFI/LRA MFI ratios for each recipient using the formula cutoffRatio = minRatio + (maxRatio – minRatio)*(1 – 0.01). An example for each approach is provided in **Supplementary Table 2**.

In parallel, the donor-mismatched HLA-derived PIRCHE-II peptides that can be presented by recipient HLA class II were identified in a similar way as described, using the HLA typing of the donor and the HLA class II typing of the recipient as input for the PRICHE-II algorithm. PIRCHE-II scores were determined by calculating the sum of all estimated PIRCHE-II peptides.

### Identification of Overlapping PIRCHE-II Peptides

The number of theoretical shared T-cell epitopes were identified for each recipient by comparing the PIRCHE-II peptides originating from the initial LSA-identified immunizing HLA (immunizers) with the PIRCHE-II peptides originating from the donor’s HLA typing (recall epitopes). First, duplicate PIRCHE-II peptides originating from the donor’s HLA presented by the same HLA-DRB1 molecule were merged and their weights were summed. Subsequently, for each recipient, the PIRCHE-II 9-meric peptides – representing the core peptide presented by HLA class II (48) – presented by one of the two HLA-DRB1 molecules in the immunizers dataset were compared with the PIRCHE-II 9-meric peptides presented by that same HLA-DRB1 molecule in the recall epitopes dataset. In a similar way, the shared PIRCHE-II peptides presented by the second HLA-DRB1 allele were identified. For the recipients with two identical HLA-DRB1 molecules, shared T-cell epitopes presented by only one of the two identical HLA-DRB1 alleles were considered. When a PIRCHE-II peptide was present in both the immunizers and the recall epitopes dataset, the weight as calculated in the recall epitopes dataset was allocated to that peptide. In case of no overlap, the peptide was excluded from further analysis. Next, duplicate PIRCHE-II peptides originating from different positive LSA beads presented by the same HLA-DRB1 were removed for each recipient. Finally, the sum of the weight of all identified shared PIRCHE-II peptides was calculated to get a PIRCHE-II overlap score for each recipient. The script used to identify the overlapping PIRCHE-II peptides is available on https://github.com/EPeereboom/PIRCHE-II-Overlap/blob/main/DetermineOverlappingPirchePeptides.py.

### Identification of HLAMatchmaker Eplets

To investigate the impact of B-cell epitopes on the clinical outcome, B-cell epitope mismatches for HLA-A, -B, -C, -DRB1, and -DQB1 were determined for each donor/recipient combination as described before using the HLAMatchmaker algorithm (16) (version 2.0, which takes both antibody-verified and non-antibody-verified eplets into account, available to registered PIRCHE users via https://www.pirche.com/pirche/#/pirche/matchmaker/multi/matching/request). The HLA typing of the donor and the HLA typing of the recipient with the non-extrapolated HLA-DRB1 alleles was used as input for the algorithm. HLAMatchmaker scores were determined by calculating the sum of all potential B-cell epitopes mismatches.

### Statistical Analysis

Statistical analyses were performed using R version 4.0.3 (R Core Team, Vienna, Austria). The above-described four approaches to positively select the theoretical immunizing HLA alleles were evaluated by determining the area under the curve (AUC, ‘pROC’ package version 1.18.0) for each method. In addition, the median ln-transformed PIRCHE-II overlap scores (ln(PIRCHE-II overlap score + 1) – or Shared T-cell EPitopes (STEP) scores – of recipients who did and recipients who did not experience graft failure during the 10-year follow-up were compared using Mann-Whitney U tests.

The effect of recipient age, recipient sex, donor age, donor sex, year of transplantation, historic peak panel reactive antibodies (hPRA), repeat transplant, type of donation [donation after brain death (DBD)/donation after circulatory death (DCD)], cold ischemic period, number of HLA-A/B/DR mismatches, HLAMatchmaker score, ln(PIRCHE-II score + 1), and ln(PIRCHE-II overlap score + 1) on the 10-year risk of death-censored kidney graft failure were studied using an univariate Cox proportional hazards analysis (‘survival’ package version 3.2-13). In addition, a multivariable Cox proportional hazards analysis with all univariately studied variables was performed using a stepwise variable selection method alternating between forward and backward selection (‘My.stepwise’ package version 0.1.0). Significance Levels for Entry (SLE) and Stay (SLS) were set to p = 0.15. For both univariate and multivariable Cox models, repeat transplant and type of donation were considered as categorical variables (references: first transplant and DCD, respectively). All other variables were treated as continuous variables. The proportional hazards assumption was checked for all variables using the Schoenfeld residuals against the transformed time (cox.zph function, ‘survival’ package). When proportional hazards could not be assumed for a variable, a time-dependent covariate was constructed using the time-transforming functionality within the survival package in R.

To study the cumulative death-censored graft failure incidence among recipients with a low and high STEP score, the study cohort was separated in two groups. The optimal cut-off value for division was determined by maximizing the Youden Index (J = sensitivity + specificity - 1), using a non-parametric approach based on kernel smoothing (‘cutpointr’ package version 1.1.1). Following division, the baseline characteristics for the two groups were compared using Mann Whitney U tests for continuous variables and chi-square tests for categorical variables. Kaplan Meier curves were constructed to depict the cumulative 10-year death-censored graft failure incidence between the two groups. When the survival graphs of the two groups crossed, an ABS permutation test (49) (‘ComparisonSurv’ package version 1.0.9) was applied to compare the difference in 10-year death-censored graft failure incidence between the groups. To exclude a bias of the HLAMatchmaker and PIRCHE-II score on the difference between the two groups, a multivariable Cox proportional hazards analysis was performed with the STEP score as a categorical variable (≤ 0.21 and > 0.21), the number of HLA-A/B/DR mismatches, the HLAMatchmaker score, and the ln-converted PIRCHE-II score, in which all variables were forced into the model.

To investigate the effect of the STEP score on early and late graft failure, additional hazard ratios (HRs) per PIRCHE-II were calculated at different virtual time points after transplantation, and among patients who did not experience graft failure before different time points. All covariates implemented in the initial multivariable model were considered when calculating these HRs. Death was considered a censoring event in the analyses. p values <0.05 were considered to be statistically significant.

## Results

### Baseline Characteristics

This study included 190 recipients who were transplanted with a kidney from a deceased donor between 1995 and 2005 and who did not have pre-transplant DSA, but had antibodies against other HLA antigens. **Table 1** describes the baseline characteristics of all transplantations included. A total of 54 kidney grafts were donated after circulatory death (DCD). The other 136 transplantations were executed following donation after brain death (DBD). The median number of HLA mismatches at serological broad level for HLA-A, -B and -DR was 2 (IQR: 2), the median number of B-cell epitopes as reflected by the HLAMatchmaker score was 24.4 (IQR: 20.5), and the median number of T-cell epitopes as reflected by the PIRCHE-II score was 49.4 (IQR: 45.8). The historic peak panel reactive antibody (hPRA) of all recipients did not exceed 75% (median: 0, IQR: 5). Consequently, all donations were allocated *via* the regular Eurotransplant Kidney Allocation System (50). After a follow-up of 10 years, 32 recipients experienced kidney graft failure. A total of 33 recipients died with a functioning graft.

**Table 1:** Baseline characteristics (n=190).

Donor age (years, median, IQR)	42.5 (25)
Donor sex (female, n, %)	83 (43.7)
Recipient age (years, median, IQR)	47.0 (18.8)
Recipient sex (female, n, %)	69 (36.3)
Year of transplantation (median, IQR)	2000 (6)
Panel reactive antibodies (%, median, IQR, range)	0 (5, 0-75)
Repeat transplant (n, %)	13 (6.8)
Type of donation (donation after circulatory death, n, %)	54 (28.4)
Cold ischemic period (hours, median, IQR)	21.0 (10.1)^a^
HLA-A/B/DR mismatches (median, IQR)	2 (2)
HLAMatchmaker score (median, IQR)	24.4 (20.5)
PIRCHE-II score (median, IQR)	49.4 (45.8)

a 6 missing values (n=184).

### Inclusion of the LSA Beads With the Highest Corrected MFI Value Distinguishes Recipients With and Without Graft Failure

**Figure 2:**
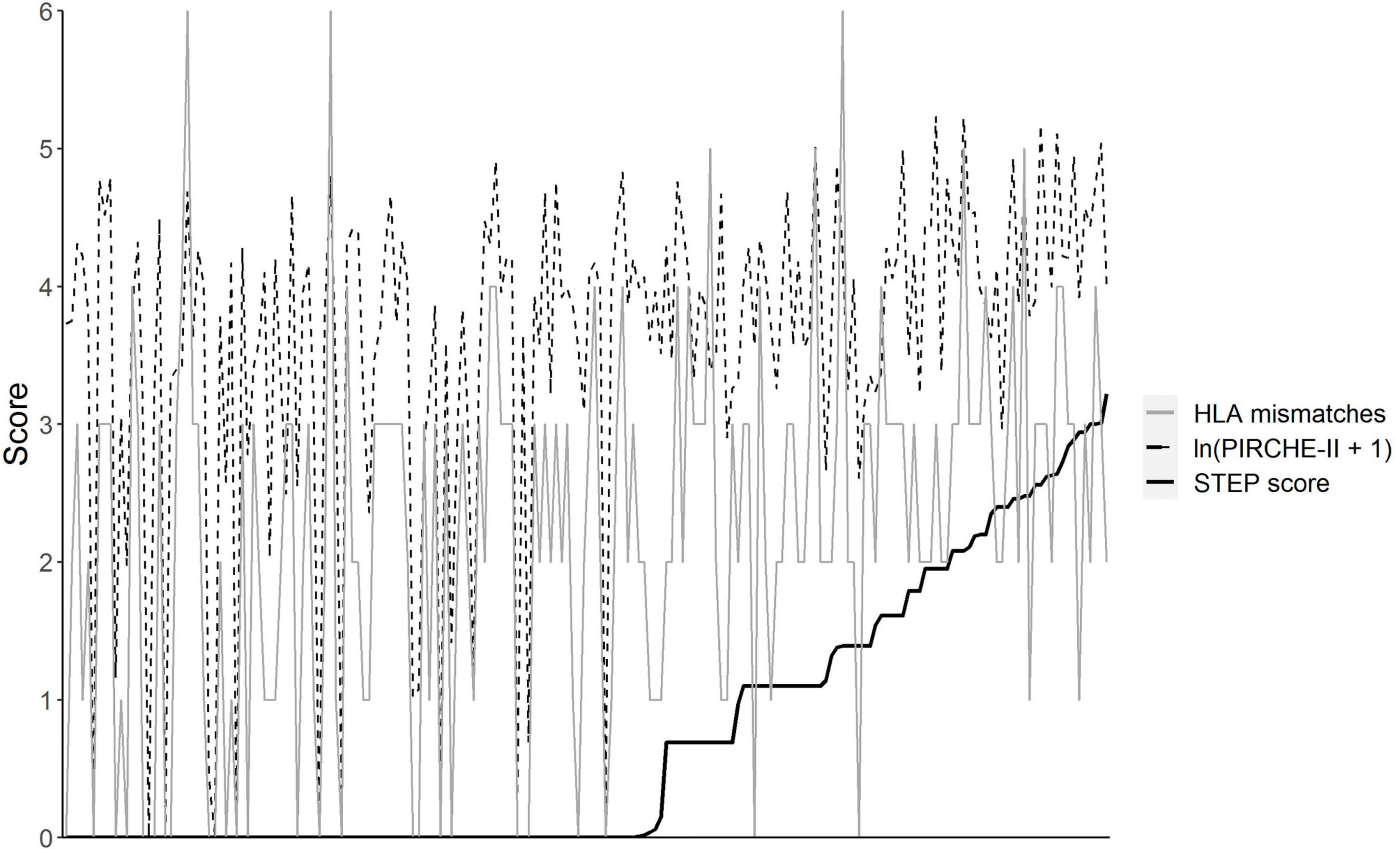
The number of HLA-A/B/DR mismatches, the ln(PIRCHE-II + 1) score, and the STEP score of all donor/recipient couples included in this study (n = 190). Donor/recipient couples have been sorted based on increasing STEP score.

To identify the theoretical immunizing HLA alleles for each recipient, the positive beads of the pre-transplant LSA assays were selected. To this end, four different approaches as described in the *Materials and Methods* section to separate the negative from the positive beads were evaluated (**Supplementary Table 2**). For each approach, the total load of overlapping PIRCHE-II epitopes was calculated for each recipient. These scores were ln-transformed (ln(PIRCHE-II overlap score + 1)) as it is known from literature that PIRCHE-II scores are logarithmically associated with the incidence of *de novo* DSA post-transplantation (31, 32). These ln-transformed PIRCHE-II overlap score are further designated as Shared T-cell EPitopes (STEP) scores. The discrepancy between the number of HLA mismatches, the ln-converted PIRCHE-II score, and the STEP score suggest that these scores are not necessarily associated with each other; for example, a higher PIRCHE-II score does not always result in a higher STEP score (**Figure 2**).

**Figure 3:**
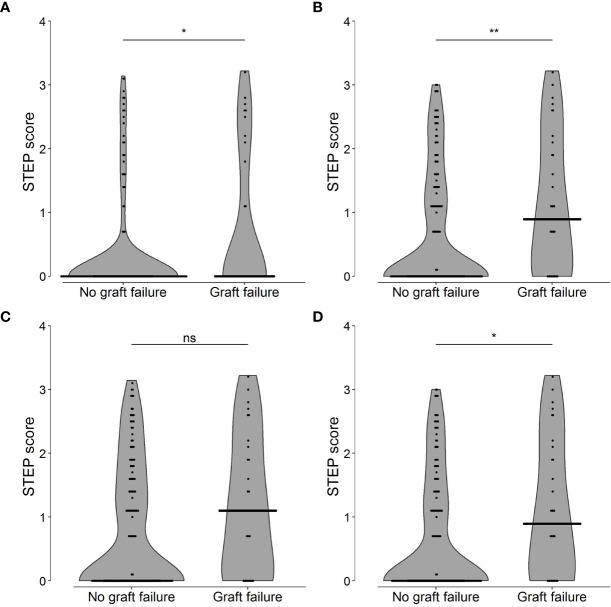
Shared T-cell EPitopes (STEP) scores of recipients with (n=32) and without graft failure (n=158) using four different approaches **(A–D)** to select the positive Luminex Single Antigen (LSA) beads. **(A)** Inclusion of the LSA beads with a mean fluorescence intensity (MFI)/MFI of the Lowest Ranked Antigen (LRA MFI) ratio higher than 5. p = 0.017, area under the curve (AUC) = 0.594. **(B)** Inclusion of the LSA bead with the highest MFI for each recipient. p = 0.010, AUC = 0.632. **(C)** Inclusion of the LSA beads falling in the upper 5% of the range of corrected MFI values for each recipient. p = 0.068, AUC = 0.596. **(D)** Inclusion of the LSA beads that fell in the upper 1% of the range of corrected MFI values for each recipient. p = 0.014, AUC = 0.627. Horizontal lines represent the median STEP scores. ROC curves with AUCs are provided in **Supplementary Figure 1**. *p < 0.05, **p < 0.01, ns, not significant.

The median STEP scores were compared between the recipients who did and the recipients who did not experienced graft failure during the 10-year period of follow-up (**Figure 3**). The approach of including the bead with the highest corrected MFI value for each recipient (approach B, **Figure 3B**) best distinguished the recipients with graft failure from the recipients without graft failure (p = 0.010, AUC: 0.632). Hence, approach B was used to select the theoretical immunizing HLA alleles needed for further analyses.

### The STEP Score Significantly Associates With Death-Censored Kidney Graft Failure in a Univariate And Multivariable Analysis

The effect of the STEP score on the 10-year risk of death-censored kidney graft failure was investigated using a univariate Cox proportional hazard analysis. In addition to the STEP score, the following variables were included in the univariate analysis: donor age, donor sex, recipient age, recipient sex, year of transplantation, hPRA, repeat transplant, type of donation (DCD/DBD), cold ischemic period, number of HLA-A/B/DR mismatches, HLAMatchmaker score, and ln-transformed PIRCHE-II score. The STEP score was shown to be significantly associated with an increased 10-year risk of graft failure censored for death (HR: 1.48, 95% CI: 1.08-2.03, p = 0.015). The HLAMatchmaker score and the ln-converted PIRCHE-II score were associated with death-censored graft failure with an HR of 1.31 (p = 0.033) and an HR of 1.43 (p = 0.082), respectively. All other tested variables were not significantly associated with transplant outcome (**Table 2**).

**Table 2:** Univariate and multivariable Cox proportional hazards analysis of the effect of donor age, donor sex, recipient age, recipient sex, year of transplantation, historic peak panel reactive antibodies (hPRA), repeat transplant, type of donation [donation after brain death (DBD) or donation after circulating death (DCD)], cold ischemic period, number of HLA-A/B/DR mismatches, HLAMatchmaker score, ln(PIRCHE-II score + 1), and Shared T-cell EPitopes (STEP) score on the 10-year risk of death-censored graft failure.

	Univariate analysis			Multivariable analysis (stepwise variable selection)		
	HR	95% CI	p value	HR	95% CI	p value
Donor age (years)	1.01	0.99-1.04	0.239			
Donor sex (ref: male)	1.01	0.50-2.04	0.979			
Recipient age (years)	1.00	0.98-1.02	0.946			
Recipient sex (ref: male)	0.46	0.20-1.07	0.073			
Year of transplantation (years)	0.92	0.82-1.03	0.155			
Historic peak panel reactive antibodies (percentage)	1.00	0.97-1.03	0.873			
Repeat transplant (ref: first transplant)	0.89	0.21-3.71	0.860			
Type of donation (ref: DCD)	1.40	0.67-2.90	0.367			
Cold ischemic period (hours)	1.04	1.00-1.09	0.078			
Number of HLA-A/B/DR mismatches	1.20	0.94-1.54	0.141			
HLAMatchmaker score (per 10 increment)	1.31	1.02-1.68	**0.033**			
ln(PIRCHE-II score + 1)	1.43	0.96-2.12	0.082			
STEP score	1.48	1.08-2.03	**0.015**	1.48	1.08-2.03	**0.015**

For each variable, the proportional hazards assumption was tested using the Schoenfeld residuals. For year of transplantation, proportional hazards could not be assumed. Therefore, a time-dependent covariate was constructed from this variable. Displayed in the table is the hazard ratio (HR) of each variable with the 95% confidence interval (CI) and the p value. In the multivariable Cox proportional hazards model, only the STEP score was included in the model. Consequently, the HRs of the other variables in the multivariable analysis are not provided. Bold values indicate a significant difference (p < 0.05).

To further investigate these findings and to correct for possible confounders, a multivariable Cox proportional hazard analysis was performed using a stepwise variable selection method. All variables that were univariately analyzed were included in the multivariable analysis as well. Only the STEP score was significantly associated in the multivariable model with death-censored graft failure with a hazard ratio of 1.48 (95% CI: 1.08-2.03, p = 0.015), meaning that with a one-unit increase of the STEP score, the 10-year risk for kidney graft failure increases with 48%. Thus, the STEP score was observed to have a significant effect on the 10-year risk of death-censored kidney graft failure in the univariate as well as in the multivariable Cox proportional hazard analysis.

### Recipients With a High STEP Score Have a Higher Cumulative 10-Year Death-Censored Graft Failure Incidence as Compared to Recipients With a Low STEP Score

**Figure 4:**
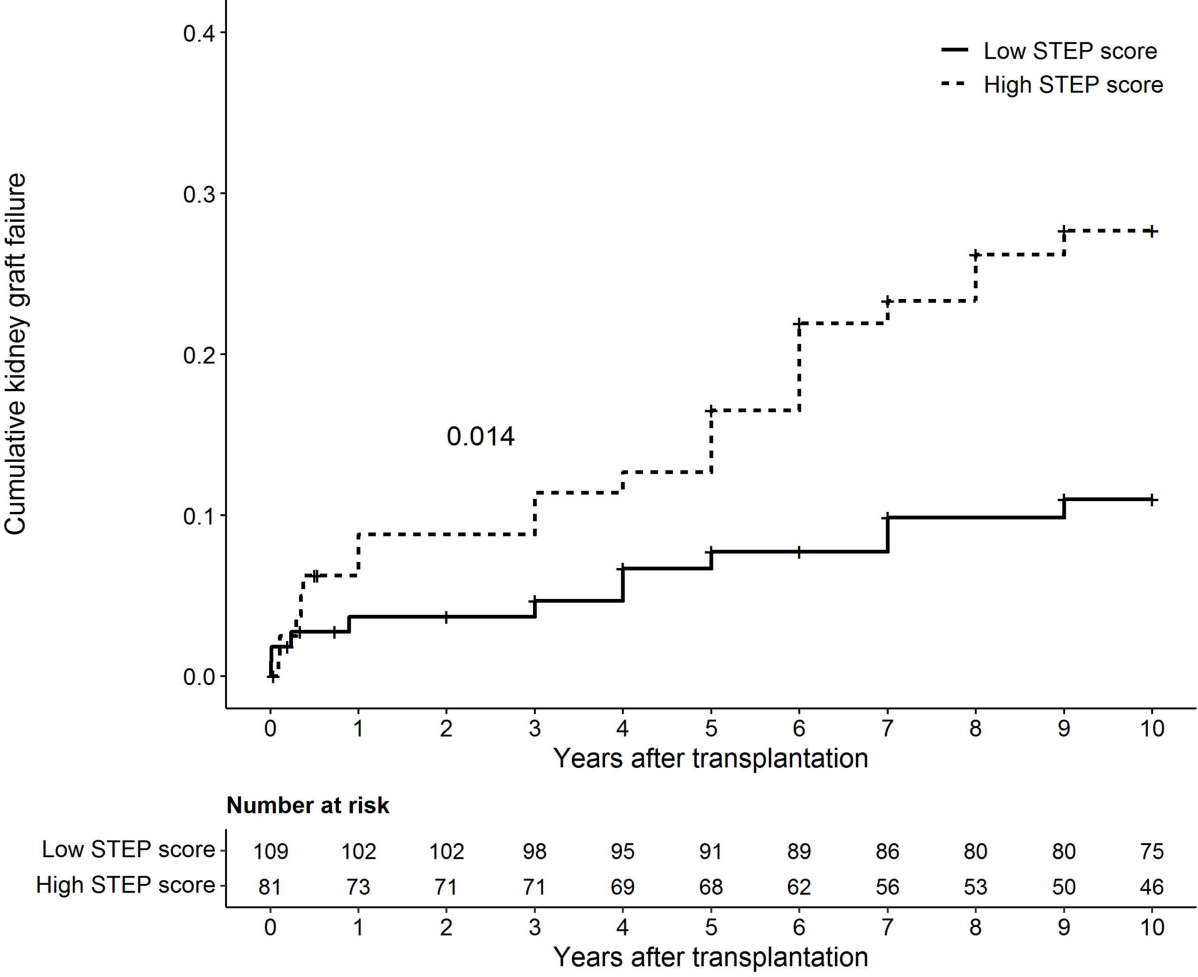
Cumulative 10-year death-censored kidney graft failure incidence among recipients with a low Shared T-cell EPitopes (STEP) score (≤ 0.21, n = 109, 11 events during follow-up) and recipients with a high STEP score (> 0.21, n = 81, 21 events during follow-up). p = 0.014.

To visualize the effect of shared PIRCHE-II epitopes on the cumulative 10-year risk of death-censored graft failure, the study population was divided into a group with a low and with a high STEP score. Based on the optimal Youden Index, a cut-off value of 0.21 was applied (J = 0.277). Comparison of the baseline characteristics of the two recipient groups showed that the group with a STEP score higher than 0.21 (n = 81) had a significantly higher number of HLA-A/B/DR mismatches (p < 0.001) and a higher HLAMatchmaker (p < 0.001) and ln-converted PIRCHE-II score (p < 0.001) than the group with a STEP score smaller than or equal to 0.21 (n = 109) (**Table 3**). Other variables did not differ significantly between the two groups. To compare the incidence of kidney graft failure among the recipients with a low and with a high STEP score, the cumulative death-censored kidney graft failure incidence of these two groups was examined in a Kaplan Meier analysis. Due to crossing survival graphs, an ABS permutation test was performed (49), showing a significant difference in cumulative 10-year death-censored kidney graft failure incidence between the two groups (p = 0.014) (**Figure 4**). To exclude a bias of the number of HLA mismatches, the HLAMatchmaker score and the PIRCHE-II score on the difference between the two groups, a multivariable Cox proportional hazards analysis with the number of HLA-A/B/DR mismatches, the HLAMatchmaker score, ln(PIRCHE-II + 1), and the STEP score as a categorical variable (≤ 0.21 and > 0.21) was performed in which all four variables were forced in the model (**Supplementary Table 3**). The categorized STEP score was the only significant variable in the model (p = 0.042), with an HR of 2.24 (95% CI: 1.02-4.89). Thus, these data illustrate that recipients with a higher STEP score have an increased risk of developing kidney graft failure.

**Table 3:** Baseline characteristics of recipients with a low STEP score (≤ 0.21) and recipients with a high STEP score (> 0.21).

Characteristics	Low STEP score (n=109)	High STEP score (n=81)	p value
Donor age (years, median, IQR)	42 (26)	43 (24)	0.730
Donor sex (female, n, %)	50 (45.9)	33 (40.7)	0.483
Recipient age (years, median, IQR)	48 (22)	47 (16)	0.705
Recipient sex (female, n, %)	39 (35.8)	30 (37.0)	0.860
Year of transplantation (median, IQR)	1999 (5)	2001 (6)	0.111
Panel reactive antibodies (%, median, IQR, range)	0 (5, 0-55)	0 (5, 0-75)	0.881
Repeat transplant (n, %)	10 (9.2)	3 (3.7)	0.142
Type of donation (donation after circulatory death, n, %)	27 (24.8)	27 (33.3)	0.197
Cold ischemic period (hours, median, IQR)	22.0 (10.0)^a^	20.3 (9.2)^b^	0.344
Number of HLA-A/B/DR mismatches (median, IQR)	2 (2)	3 (1)	**<0.001**
HLAMatchmaker score (median, IQR)	19.1 (19.0)	28.5 (16.2)	**<0.001**
PIRCHE-II score (median, IQR)	41.7 (45.7)	57.6 (50.2)	**<0.001**

a 2 missing values (n=107).

b 4 missing values (n=77).

p values in bold indicate a significant difference (p < 0.05).

### The STEP Score Remains Associated With Death-Censored Kidney Graft Failure Over Time and Independent of Early Graft Failures

**Figure 5:**
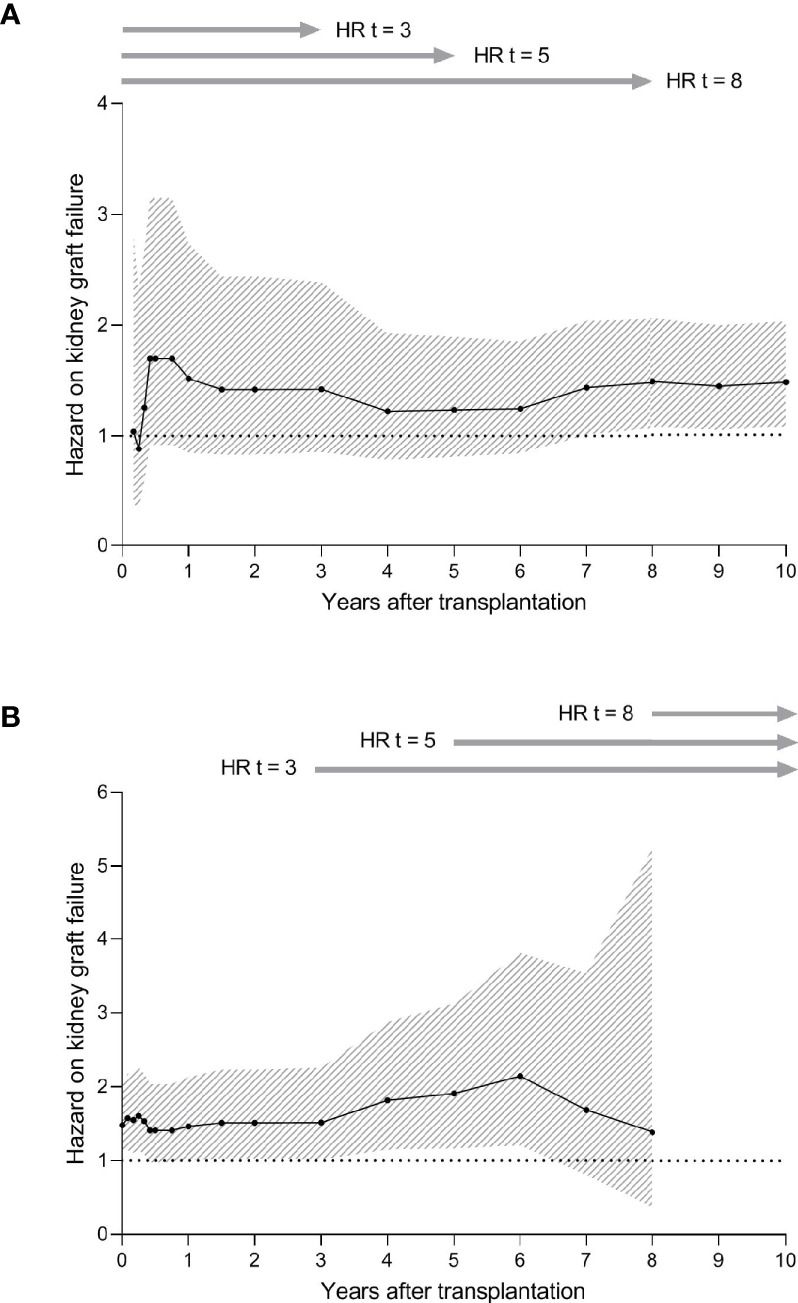
Hazard ratios (HRs) and 95% confidence intervals (CIs) for the effect of the Shared T-cell Epitopes (STEP) score on death-censored kidney graft failure. **(A)** HRs and 95% CIs for the STEP score on death-censored kidney graft failure at different virtual endpoints after transplantation (2-3-4-5-6-9-12-18 months and 2-3-4-5-6-7-8-9-10 years after transplantation). For all time spans, the number of recipients included in the analysis is 190. Due to the low number of events at 1 month transplantation, the HRs for this time span could not be calculated. **(B)** HRs and 95% CIs for the STEP score on death-censored kidney graft failure among patients who did not experience graft failure before different time points (1-2-3-4-5-6-9-12-18 months and 2-3-4-5-6-7-8 years after transplantation). These analyses were performed by setting the graft survival to 100% at each time point and by consequently calculating the HR and 96% CIs at 10 years after transplantation. The number of recipients at risk for each time span is shown in **Supplementary Table 4**. Due to the low number of events at 9 and 10 years after transplantation, the HRs for these time spans could not be calculated. HRs were calculated considering the covariates implemented in the initial multivariable model (STEP score). For both graphs, the black line represents the HR and the grey area represents the 95% CI.

Next, we investigated whether the STEP score had an effect on early or late failure (**Figure 5**). To this end, the HRs for the STEP score on death-censored kidney graft failure were calculated at different virtual endpoints after transplantation as described before (32). At 5 months post-transplantation, a peak in HR was observed (HR: 1.70, 95% CI: 0.92-3.14). After the first year, the HR declined to an HR of around 1.2 at 6 years post-transplantation and stabilized again at 7 years after transplantation (**Figure 5A**). Overall, the STEP score seemed to remain associated with the risk of death-censored kidney graft failure over the follow-up period of 10 years (**Figure 5A**).

To evaluate the influence of the PIRCHE-II overlap score on graft failure independent of the patients with early graft failure, the effect of the STEP score on death-censored kidney graft failure at different time points was calculated among patients who did not develop graft failure before those time points (32). After an initial peak in the first 6 months, the HR increased over time to approximately 2.1 at 6 years after transplantation (**Figure 5B**). After these 6 years, the HR seemed to decline to an HR of 1.4 at 8 years after transplantation. Thus, the observed trend in our data suggests that the effect of the STEP score on the risk of death-censored graft failure is most prominent in the first year and around the sixth year after transplantation (**Figure 5A**). In addition, it is suggested that also among recipients who do not experience graft failure during the early period, STEP remains associated with graft failure.

## Discussion

Memory is a characteristic aspect of the adaptive immune system. While memory is of utterly beneficial importance in vaccinology and tumor immunology, recall responses significantly hamper a positive outcome in transplantation (51–53). Although much is known about B-cell memory due to pre-transplant DSA (51, 52), the knowledge about T-helper-cell memory in the context of organ transplantation remains limited. As a surrogate marker for donor-reactive memory T-helper cells, we here evaluated whether the potential presence of T-cell epitopes shared between immunizing and recall HLA would affect the risk of kidney graft failure. We observed a significant association between the predicted T-cell epitope overlap and the 10-year risk of death-censored graft failure. These observations support the finding from a recent study that recipients with shared T-cell epitopes have higher early *de novo* DSA formation as compared to patients without shared T-cell epitopes (41). Our results may provide an explanation for why recipients with pre-transplant non-donor-specific HLA antibodies have a decreased graft survival (39) and diminished graft function (40) as compared to patients without any HLA antibodies. Possibly, recipients with non-donor-specific HLA antibodies have pre-transplant donor-HLA reactive CD4^+^ T-helper cells.

The comparison between recipients with a low and a high STEP score showed that the group with theoretically more shared T-cell epitopes had a significant higher cumulative death-censored kidney graft failure incidence during follow-up. A significant difference in the number of HLA mismatches, the HLAMatchmaker score, and the PIRCHE-II score between the two groups was observed, which might be due to the relation between the STEP score, the number of HLA mismatches, the PIRCHE-II score, and the HLAMatchmaker score. The observed differences could indicate that the difference in graft failure incidence might be due to primary immune responses. However, a multivariable Cox proportional hazards analysis showed that the categorical STEP score was the only variable with a substantial and significant effect on death-censored graft failure (HR: 2.24, p = 0.042). Thus, these findings suggest that the difference in cumulative incidence of 10-year graft failure are mainly a consequence of the recall response and not only due to *de novo* immune responses induced by the transplantation. However, due to the limited sample size and the low number of graft losses in this cohort, these findings need to be confirmed in a bigger cohort. Then, also stratification analyses could be performed to further ensure exclusion of all potential confounding effects.

A higher STEP score may be associated with graft failure mainly in the first months after transplantation due to the pre-existing donor-reactive CD4^+^ memory T-helper cells. Our results indicate that the amount of potential T-cell epitopes shared between immunizing HLA and donor HLA might have an effect on graft failure mainly in first year and around 6 years after transplantation, with the biggest effect being present shortly after transplantation (**Figure 5A**). Possibly, the observed effect in first year post-transplantation is due to the presence of donor-reactive CD4^+^ memory T-helper cells, while the observed effect in the later years may be the consequence of a higher PIRCHE-II score and HLAMatchmaker score in patients with a higher STEP score. Further research including interaction analyses should be performed in a bigger cohort to investigate the role of PIRCHE-II and HLAMatchmaker in the context of the STEP score in greater detail.

If donor-reactive CD4^+^ memory T-helper cells indeed lead to more graft failure, this could potentially be due to a faster B-cell expansion and earlier class switching as compared to naive alloimmune T-helper cells, leading to a more rapid production of DSA (34). This notion is supported by the finding that the amount of potential T-cell epitopes shared between donor and recipient HLA are linked with *de novo* DSA development (41). In addition, mice with pre-transplant donor-reactive CD4^+^ memory T-helper cells have been shown to rapidly develop antibody-mediated graft rejection (54). Besides humoral response, these CD4^+^ memory T-helper cells could also have an impact on the cellular response (18, 19). However, due to the lack of data regarding the cause of graft failure and *de novo* DSA development in the current cohort, we were not able to make conclusions about the effect of the STEP score on the risk of rejection. The PROCARE cohort recorded graft failure as transplant outcome and contained limited information on rejection; since biopsies were not always performed, the registered rejections in the cohort were not biopsy-proven. It therefore remains to be validated whether patients with T-cell epitopes shared between immunizing and donor HLA have an increased risk for graft failure as a consequence of a higher risk of antibody-mediated rejection.

HLA typing data for retrospective epitope studies is generally limited by the level of typing. Although nowadays, patients are more frequently being typed at the allelic level using NGS, donor and recipient high-resolution HLA typing was not yet available in the current study. Since high-resolution HLA typing is essential for the precise identification of B- and T-cell epitopes, the high-resolution HLA alleles were imputed from the available serological HLA typing as described (46). This method of extrapolation is more reliable than choosing the highest frequent high-resolution HLA allele that is present in the general population, because it also includes the less-frequent HLA alleles (46). However, in the context of the present study, this approach does not allow the PIRCHE-II algorithm to define by which high-resolution HLA-DRB1 the computed PIRCHE-II peptide is presented and, consequently, to compare the calculated PIRCHE-II peptides originating from the immunizing and donor HLA. To allow the identification of the overlapping PIRCHE-II peptides, the most likely high-resolution HLA-DRB1 of each recipient was selected based on each recipient’s serological HLA-DR. In our study, only recipients from whom the high-resolution HLA-DRB1 could be imputed with at least 65% certainty were included. Still, the uncertainty of the extrapolation from low- to high-resolution and HLA extrapolation and the selection of the most probable high-resolution HLA-DRB1 for each patient might have led to an under- or overestimation of the effect of the STEP score on graft failure in this study. Especially when calculating the STEP score for a single donor/recipient combination, high-resolution – and ultimately allelic – HLA typing is a prerequisite, since serological HLA typing introduces noise in the identification of the PIRCHE-II peptides and their overlap.

Another limitation regarding HLA typing data for retrospective epitope studies is the number of typed HLA loci. In this study, HLA typing was available for HLA-A, -B, and -DR, and HLA-C and -DQ when available. However, PIRCHE-II peptides could also originate from other HLA loci. For future studies, it could be of interest to do locus-specific analyses to assess whether certain loci have a larger impact on the STEP score as compared to others. In addition, only HLA-DR was considered as a restriction element in this study; because it has been assumed that HLA-DR is the most important in restriction element for antigen presentation (55–57), T-cell epitope prediction is generally focused on HLA-DR. Therefore, the accuracy of the T-cell epitope prediction in the context of HLA-DQ and -DP is often limited. Still, T-cell epitopes could potentially also be presented by other HLA class II antigens, but the relevance of peptide presentation by these other loci has yet to be demonstrated.

The initial immunizing HLA of the recipients included in this study was determined based on serum reactivity, i.e. the specificity of the recipients’ pre-transplant anti-HLA antibodies. This approach might have led to artifacts. For example, HLA alleles that are sharing B-cell epitopes with the immunizing HLA, but were not inducing the primary immunizing response, might be included as immunizing HLA in the analyses, resulting in an overestimation of the potential T-cell epitopes. In addition, research has shown that CD4^+^ memory T-helper cells specific to one alloantigen might provide help to naïve B cells specific for another alloantigen, representing an unlinked system of allorecognition (58). This could imply that for some patients, the T-cell epitope originates from another alloantigen as the T-cell epitope as identified using the concept of linked recognition. Moreover, the immunizing event might have been fully T-cell mediated, with T-cell epitopes not having resulted in HLA antibodies during the initial immunization. Finally, the T-cell epitope could theoretically have originated from one polymorphic chain of a heterodimeric HLA molecule, while the B-cell epitope could have originated from the other polymorphic chain. This option applies only to the HLA-DQ and -DP heterodimers, since HLA-DRA1 is not polymorphic at the protein level (59). Consequently, the T-cell epitope originating from the immunizing HLA-DQ or -DP might be on a different polymorphic chain than the polymorphic chain identified with the LSA assay. As a result, this recipient could have had an overlapping PIRCHE-II peptide with the donor HLA while this was not predicted by the PIRCHE-II algorithm. Having information about the immunizing event could give more certainty about the immunizing HLA and might therefore lead to a more reliable STEP prediction.

In conclusion, we have shown that the STEP score is strongly associated with the 10-year risk of kidney graft failure, confirming that shared T-cell epitopes and pre-transplant CD4^+^ donor-reactive memory T-helper cells may play an important role in the development of graft failure. Potentially, this approach could be useful for monitoring patients following a kidney transplantation. However, because of the limited sample size and the serological HLA typing, our findings need to be validated in a bigger cohort with high-resolution HLA typing. In addition, since the identification of the T-cell epitopes shared between immunizing and donor HLA is relatively complex in the current setting as the immunizing T-cell epitopes are calculated by means of the antibody reactivity and the concept of linked recognition, the feasibility of this approach remains to be investigated.

## Data Availability Statement

The data analyzed in this study is subject to the following licenses/restrictions: Usage of dataset is limited by the informed consent and ethical committee permission. Requests to access these datasets should be directed to h.g.otten@umcutrecht.nl.

## Ethics Statement

The studies involving human participants were reviewed and approved by Biobank Research Ethics Committee (TCBio), University Medical Center Utrecht. Written informed consent to participate in this study was provided by the participants’ legal guardian/next of kin.

## Author Contributions

All authors met the authorship criteria as described by *Frontiers in Immunology*. EP, BM, TT, MN, JD, TK, AZ, KG, and ES were involved in the design of the work and interpretation of the data. IJ, WA, AM, LH, MCB, FR, MV, EK, MS, JS, BH, AL, LB, CR, MT, CV, LW, ED, MG, MC, FI, AN, NL, WS, KP, NW, IB, FB, AV, JF, MGHB, DR, FC, HO, SH, AZ, and ES were involved in the acquisition of the data. All authors were involved in drafting or revising the manuscript and approved the final version. All authors agree to be accountable for all aspects of the work in ensuring that questions related to the accuracy or integrity of any part of the work are appropriately investigated and resolved.

## Funding

This study was supported by research funding from the Dutch Kidney Foundation project code CP12.23 "Risk assessment of kidney graft failure by HLA antibody profiling" and project code CP1801 “Prediction of Kidney Graft Survival by Immune Profiling - Towards Clinical Application in Personalized Medicine”, by research funding from EU Horizon 2020 for the ULISES project, code 899708, and by research funding from the International HLA and Immunogenetics Workshop Foundation.

## Conflict of Interest

The authors of this manuscript have conflicts of interest to disclose. The UMC Utrecht has filed a patent application on the prediction of an alloimmune response against mismatched HLA. ES is listed as inventor on this patent. MN is employed by PIRCHE AG, which publishes the PIRCHE web-portal.

The remaining authors declare that the research was conducted in the absence of any commercial or financial relationships that could be construed as a potential conflict of interest.

## Publisher’s Note

All claims expressed in this article are solely those of the authors and do not necessarily represent those of their affiliated organizations, or those of the publisher, the editors and the reviewers. Any product that may be evaluated in this article, or claim that may be made by its manufacturer, is not guaranteed or endorsed by the publisher.

## Abbreviations

DBD, donation after brain death; DCD, donation after circulatory death; DSA, donor-specific antibodies; HLA, human leukocyte antigen; hPRA, historic peak panel reactive antibodies; HR, hazard ratio; LRA, lowest ranked antigen; LSA, Luminex Single Antigen; MFI, mean fluorescence intensity; PIRCHE-II, Predicted Indirectly ReCognizable HLA Epitopes presented by recipient HLA class II; STEP, Shared T-cell EPitopes.
