# Supplementary material for: T-Cell Epitopes Shared Between Immunizing HLA and Donor HLA Associate With Graft Failure After Kidney Transplantation
Source: Front Immunol. 2021 Nov 18;12:784040. doi: 10.3389/fimmu.2021.784040 (PMC8637278; doi:10.3389/fimmu.2021.784040)
Supplement: Supplementary file 1 [file DataSheet_1.docx]

# Supplement

**Table 1.** Example of the recipients’ serological to high-resolution HLA-DRB1 extrapolation. Using the serological HLA typing of the recipient (serological genotype), all possible high-resolution genotypes with their weights - representing the likelihood of a specific high-resolution typing – were calculated (high-res. genotype #1-6). In this example, the most likely HLA-DRB1 alleles were HLA-DRB1*03:01 and HLA-DRB1*04:01, with a weight of 0.7781 + 0.0192 + 0.02 = 0.8173. Since this weight is above the threshold of 0.65, this recipient is considered in further analyses and the recipient’s serological HLA-DR3 and HLA-DR4 is replaced by DRB1*03:01 and HLA-DRB1*04:01.

|  | **Serological genotype** | **High-res. genotype #1** | **High-res. genotype #2** | **High-res. genotype #3** | **High-res. genotype #4** | **High-res. genotype #5** | **High-res. genotype #6** |
| --- | --- | --- | --- | --- | --- | --- | --- |
| **HLA-A** | A1 | A*01:01 | A*01:01 | A*01:01 | A*01:01 | A*01:01 | A*01:01 |
| **HLA-A** | A28 | A*68:02 | A*68:02 | A*68:01 | A*68:02 | A*68:02 | A*68:01 |
| **HLA-B** | B51 | B*51:01 | B*51:01 | B*51:01 | B*51:01 | B*51:01 | B*51:01 |
| **HLA-B** | B8 | B*08:01 | B*08:01 | B*08:01 | B*08:01 | B*08:01 | B*08:01 |
| **HLA-C** | Cw7 | C*07:01 | C*07:01 | C*07:01 | C*07:01 | C*07:01 | C*07:02 |
| **HLA-C** | Cw7 | C*07:02 | C*07:02 | C*07:01 | C*07:02 | C*07:02 | C*07:01 |
| **HLA-DRB1** | DR3 | ***DRB1*03:01*** | DRB1*03:01 | ***DRB1*03:01*** | DRB1*03:01 | DRB1*03:01 | ***DRB1*03:01*** |
| **HLA-DRB1** | DR4 | ***DRB1*04:01*** | DRB1*04:07 | ***DRB1*04:01*** | DRB1*04:08 | DRB1*04:03 | ***DRB1*04:01*** |
| **HLA-DQB1** | DQ2 | DQB1*02:01 | DQB1*02:01 | DQB1*02:01 | DQB1*02:01 | DQB1*02:01 | DQB1*02:01 |
| **HLA-DQB1** | DQ7 | DQB1*03:01 | DQB1*03:01 | DQB1*03:01 | DQB1*03:01 | DQB1*03:04 | DQB1*03:01 |
| **Weight** |  | ***0.7781*** | 0.1396 | ***0.0192*** | 0.0317 | 0.0114 | ***0.02*** |

**Table 2.** Example of the results of the LSA assay of one recipient, performed to determine the initial theoretical immunizing HLA. LSA beads are ranked from high to low MFI/LRA MFI ratio. Various approaches were evaluated to select the immunizing HLA antigens. **(1)** Approach A: Inclusion of the beads with an MFI/LRA MFI ratio higher than 5. Consequently, bead 1 (DRB3*01:01) and bead 2 (DRB3*03:01) were considered as immunizing HLA antigens. **(2)** Approach B: Inclusion of the bead(s) with the highest positive MFI. Consequently, only bead 1 (DRB3*01:01) was considered as the immunizing HLA antigen. **(3)** Approach C: Inclusion of the beads with an MFI/LRA MFI ratio in the upper 5% range of MFI/LRA MFI ratios for each recipient. The cut-off value was calculated using the formula cutoffRatio = minRatio + (maxRatio – minRatio)*(1 – 0.05). For this recipient, the minimum MFI/LRA MFI ratio was 1 and the maximum ratio was 61.03. Therefore, the cut-off value was 58.03. Consequently, only bead 1 (DRB3*01:01) was considered as the immunizing HLA antigen. **(4)** Approach D: Inclusion of the beads with an MFI/LRA MFI ratio in the upper 1% range of MFI/LRA MFI ratios for each recipient. The cut-off value was calculated using the formula cutoffRatio = minRatio + (maxRatio – minRatio)*(1 – 0.01). For this recipient, the minimum MFI/LRA MFI ratio was 1 and the maximum ratio was 61.03. Therefore, the cut-off value was 59.43. Consequently, only bead 1 (DRB3*01:01) was considered as the immunizing HLA antigen. *Abbreviations: LSA = Luminex Single Antigen; HLA = Human Leukocyte Antigen; MFI = Mean Fluorescence Intensity; LRA = Lowest Ranked Antigen.*

| **LSA bead** | **HLA antigen** | **MFI value** | **LRA MFI value** | **MFI/LRA MFI ratio** |  |
| --- | --- | --- | --- | --- | --- |
| 1 | DRB3*01:01 | 2899 | 47.5 | 61.031579 |  |
| 2 | DRB3*03:01 | 637.5 | 47.5 | 13.421053 |  |
| 3 | DPA1*02:02, DPB1*01:01 | 157.5 | 47 | 3.3510638 |  |
| 4 | DPA1*02:01, DPB1*01:01 | 143 | 47 | 3.0425532 |  |
| 5 | DPA1*01:03, DPB1*01:01 | 140 | 47 | 2.9787234 |  |
| 6 | DRB1*13:01 | 138 | 47.5 | 2.9052632 |  |
| 7 | DRB1*11:04 | 131 | 47.5 | 2.7578947 |  |
| 8 | DPA1*03:01, DPB1*01:01 | 129 | 47 | 2.7446809 |  |
| 9 | DPA1*02:01, DPB1*17:01 | 120.5 | 47 | 2.5638298 |  |
| 10 | DPA1*02:02, DPB1*04:01 | 120 | 47 | 2.5531915 |  |
| … | … | … | … | … |  |
| 82 | DPA1*01:03, DPB1*04:01 | 47 | 47 | 1 |  |
|  |  |  |  |  |  |

| **A** | 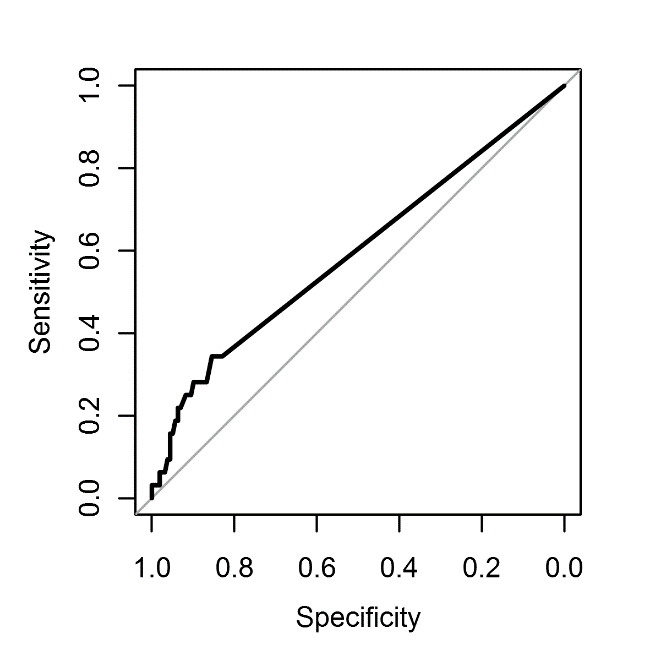 | **B** | 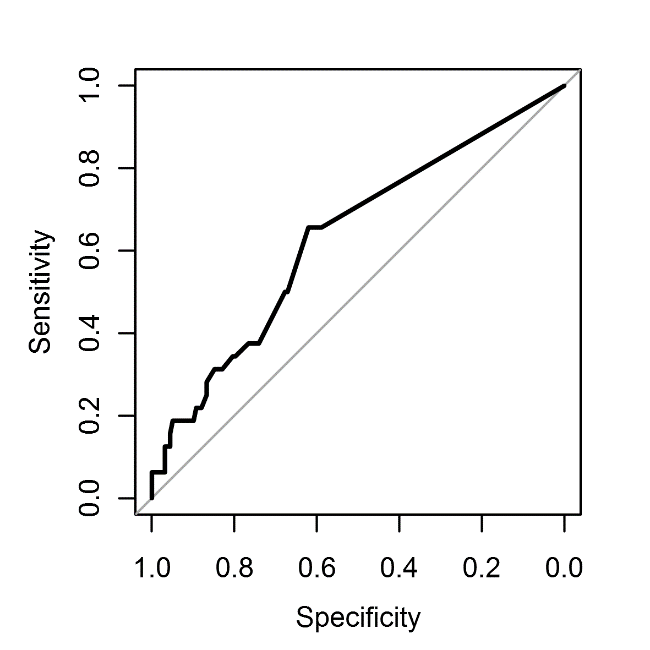 |
| --- | --- | --- | --- |
| **C** | 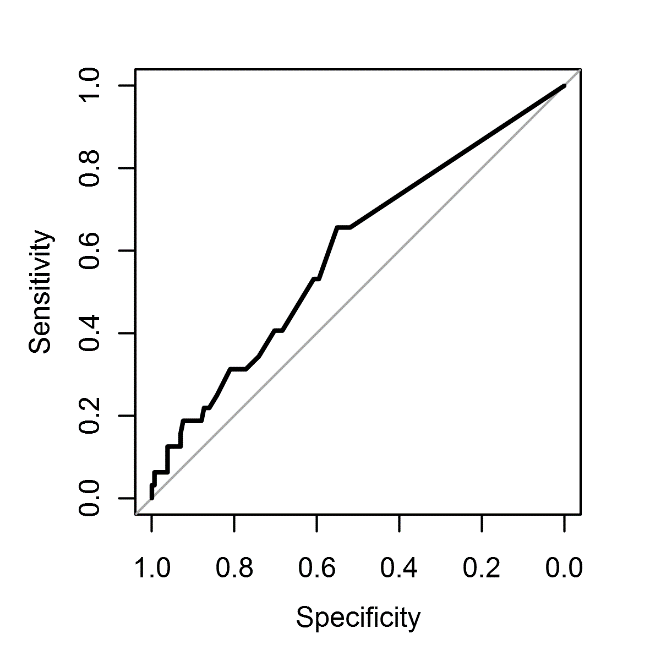 | **D** | 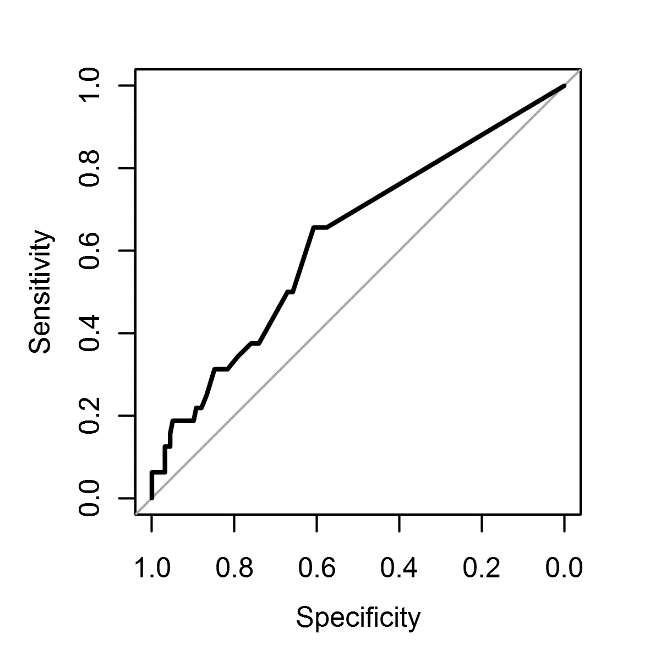 |
| **Figure 1.** Receiver Operator Curves (ROCs) for the four different approaches (A-D) to select the positive Luminex Single Antigen (LSA) beads. **(A)** Inclusion of the LSA beads with a mean fluorescence intensity (MFI)/MFI of the Lowest Ranked Antigen (LRA MFI) ratio higher than 5. Area under the curve (AUC) = 0.594. **(B)** Inclusion of the LSA bead with the highest MFI for each recipient. AUC = 0.632. **(C)** Inclusion of the LSA beads falling in the upper 5% of the range of MFI values for each recipient. AUC = 0.596. **(D)** Inclusion of the LSA beads that fell in the upper 1% of the range of MFI values for each recipient. AUC = 0.627. | | | |

**Table 3.** Multivariable Cox proportional hazards analysis of the effect of the HLAMatchmaker score, ln(PIRCHE-II + 1), and the STEP score as a categorical variable (≤ 0.21 and > 0.21) on the 10-year risk of death-censored kidney graft failure. All three variables were forced in the model (method enter). For all variables, proportional hazards could be assumed, as tested using the Schoenfeld residuals. Displayed in the table is the hazard ratio (HR) of each variable with the 95% confidence interval (CI) and the p value.

|  | **HR** | **95% CI** | **p value** |
| --- | --- | --- | --- |
| HLAMatchmaker score (per 10 increment) | 1.15 | 0.82-1.63 | 0.418 |
| ln(PIRCHE-II score + 1) | 1.09 | 0.66-1.82 | 0.733 |
| STEP score (ref: < 0.21) | 2.24 | 1.02-4.86 | 0.043 |

**Table 4.** Number of recipients at risk for developing death-censored graft failure for the different time spans following transplantation, as analyzed in Figure 4B.

| **Time after transplantation** | **Number at risk** |
| --- | --- |
| 0 months | 190 |
| 1 month | 189 |
| 2 months | 185 |
| 3 months | 183 |
| 4 months | 182 |
| 5 months | 179 |
| 6 months | 179 |
| 9 months | 176 |
| 12 months | 174 |
| 18 months | 173 |
| 2 years | 173 |
| 3 years | 168 |
| 4 years | 160 |
| 5 years | 156 |
| 6 years | 149 |
| 7 years | 141 |
| 8 years | 131 |
